# A self-compatible pear mutant derived from γ-irradiated pollen carries an 11-Mb duplication in chromosome 17

**DOI:** 10.3389/fpls.2024.1360185

**Published:** 2024-03-05

**Authors:** Sogo Nishio, Kenta Shirasawa, Ryotaro Nishimura, Yukie Takeuchi, Atsushi Imai, Nobuko Mase, Norio Takada

**Affiliations:** ^1^ Deciduous Fruit Tree Breeding Group, Division of Fruit Tree Breeding Research, Institute of Fruit Tree and Tea Science, National Agriculture and Food Research Organization, Tsukuba, Japan; ^2^ Department of Frontier Research and Development, Kazusa DNA Research Institute, Kisarazu, Japan; ^3^ Fruit Tree Smart Production Group, Division of Fruit Tree Production Research, Institute of Fruit Tree and Tea Science, National Agriculture and Food Research Organization, Higashihiroshima, Japan; ^4^ Citrus Breeding and Production Group, Division of Citrus Research, Institute of Fruit Tree and Tea Science, National Agriculture and Food Research Organization, Shizuoka, Japan

**Keywords:** *Pyrus*, S-haplotype, depth of coverage, DdRAD-seq, whole-genome sequencing, triploid

## Abstract

Self-compatibility is a highly desirable trait for pear breeding programs. Our breeding program previously developed a novel self-compatible pollen-part Japanese pear mutant (*Pyrus pyrifolia* Nakai), ‘415-1’, by using γ-irradiated pollen. ‘415-1’ carries the *S*-genotype *S_4_dS_5_S_5_*, with “*d*” indicating a duplication of *S*_5_ responsible for breakdown of self-incompatibility. Until now, the size and inheritance of the duplicated segment was undetermined, and a reliable detection method was lacking. Here, we examined genome duplications and their inheritance in 140 F_1_ seedlings resulting from a cross between ‘515-20’ (*S_1_S_3_*) and ‘415-1’. Amplicon sequencing of *S-RNase* and *SFBB18* clearly detected *S*-haplotype duplications in the seedlings. Intriguingly, 30 partially triploid seedlings including genotypes *S_1_S_4_dS_5_*, *S_3_S_4_dS_5_*, *S_1_S_5_dS_5_*, *S_3_S_5_dS_5_*, and *S_3_S_4_dS_4_* were detected among the 140 seedlings. Depth-of-coverage analysis using ddRAD-seq showed that the duplications in those individuals were limited to chromosome 17. Further analysis through resequencing confirmed an 11-Mb chromosome duplication spanning the middle to the end of chromosome 17. The duplicated segment remained consistent in size across generations. The presence of an *S_3_S_4_dS_4_* seedling provided evidence for recombination between the duplicated *S_5_* segment and the original *S_4_* haplotype, suggesting that the duplicated segment can pair with other parts of chromosome 17. This research provides valuable insights for improving pear breeding programs using partially triploid individuals.

## 1 Introduction

Self-incompatibility, a widespread phenomenon present in approximately 40% of plant species, serves as a fundamental mechanism for preventing inbreeding and preserving genetic diversity (Igic et al., 2008; Fujii et al., 2016). The Rosaceae, Solanaceae, and Plantaginaceae are presumed to share the same gametophytic self-incompatibility mechanism, which is regulated by an S-locus that carries a single pistil determinant gene and multiple pollen determinant genes (McClure, 2004). The single pistil determinant gene is commonly referred to as *S-RNase*; it inhibits pollen tube growth of the corresponding pollen genotype by its cytotoxic ribonuclease activity. On the other hand, the pollen determinant genes, which are tightly linked to *S-RNase*, are referred to as *S-locus F-box* (*SLF*) or *S-locus F-box brother* (*SFBB*): the protein products of these genes detoxify non-self S-RNase proteins. According to the collaborative non-self-recognition model, each SLF within a given *S*-haplotype recognizes a different S-RNase protein of the other *S-*haplotypes, thereby allowing pollen tubes to grow through the stylar tissue (Kubo et al., 2010; Sassa, 2016). Although self-incompatibility effectively prevents self-fertilization, thus reducing inbreeding and the risk of species extinction, it necessitates cross-pollination from different genotypes to ensure stable fruit set and production. Artificial pollination is common in Japanese pear production, although some cultivars are self-compatible (Tamura, 2006). Notably, over half of Japanese pear producers rely on imported pollen from foreign sources (Shibasaki et al., 2023). The viable timeframe for conducting artificial pollination is confined to a mere week and is contingent upon environmental variables such as wind and rainfall. Consequently, labor-intensive efforts are indispensable during the flowering period (Sakamoto et al., 2009).

The discovery of the first self-compatible Japanese pear cultivar (*Pyrus pyrifolia* Nakai), ‘Osa-Nijisseiki’, can be traced back to a bud mutant arising within ‘Nijisseiki’ (*S_2_S_4_*) in 1978. This pivotal discovery expedited the breeding of self-compatible cultivars and advanced our comprehension of the genetic underpinnings of self-compatibility in Japanese pear (Saito, 2016). Subsequent crossing experiments involving ‘Osa-Nijisseiki’ and ‘Nijisseiki’ revealed that ‘Osa-Nijisseiki’ contained a stylar-part mutant of the *S_4_* haplotype, designated *S_4_^sm^*. Sequence analysis of a bacterial artificial chromosome library revealed a deletion spanning 236 kb, encompassing a sequence from 48 kb upstream to 188 kb downstream of *S_4_-RNase* (Okada et al., 2008). Remarkably, this deleted segment contained not only the *S_4_-RNase* but also one of the *SFBB* genes. Further crossing experiments revealed that *S_4_^sm^* pollen is rejected by pistils carrying *S_4_* and *S_1_* haplotypes (Saito et al., 2012). The molecular markers designed to detect this deletion were used in pear breeding programs, and some new cultivars such as ‘Narumi’, ‘Shin-mizuki’, and ‘Shin-o’ were released (Saito, 2016; Nishio et al., 2022). However, more than half of the recent cultivars released in Japan carry *S_4_* and *S_1_* haplotypes, which hinders introducing *S_4_^sm^* into breeding programs.

A novel pollen-part mutant, ‘415-1’, was developed through pollination of non-irradiated ‘Kosui’ with γ-irradiated ‘Kosui’ (Sawamura et al., 2013). Because such pollinations would be successful only in the case of rare pollen grains containing a self-compatible mutation, we refer to this strategy as “selective pollination”. The *S*-haplotype on chromosome 17 of ‘415-1’ was found to be duplicated and the *S*-genotype was determined to be *S_4_dS_5_S_5_*, where “*d*” indicates that the preceding haplotype (here *S_4_*) is associated with a segment containing a duplication of the following haplotype (here *S_5_*) (Mase et al., 2014a). A single-pollen PCR analysis revealed that ‘415-1’ produced some heteroallelic pollen of genotype *S_4_dS_5_*, which would detoxify both S_4_-RNase and S_5_-RNase proteins and result in breakdown of self-incompatibility (Mase et al., 2014b). Although the duplicated region was determined to be larger than 2.2 cM by using simple sequence repeat (SSR) markers to identify triploid regions, its actual physical size and mode of inheritance had not been verified in these earlier studies. In addition, a reliable method to detect the *S*-haplotype duplication had not been established. Moreover, genetic analysis of chromosomes other than chromosome 17 has been notably limited, despite the potential presence of additional duplicated segments or deletions arising from γ-irradiation.

Recent advancements in next-generation sequencing technology, coupled with the availability of high-quality reference genomes, have greatly facilitated the analysis of genome duplication and the determination of marker dosage, specifically the allele frequency of single-nucleotide polymorphisms (SNPs) that can be used to infer the number of alleles at a given locus in polyploid plants (Shirasawa et al., 2017; Bourke et al., 2018). A technique known as double-digest restriction-site-associated DNA sequencing (ddRAD-seq; Shirasawa et al., 2016) can estimate SNP allele frequencies in polyploid plants, thus enabling the construction of genetic maps, genome-wide association studies, and the assessment of homozygosity in cultivars and selections (Shirasawa et al., 2017; Sumitomo et al., 2019; Onoue et al., 2022). Also, genome-sequencing-based coverage methods capable of detecting duplicated and deleted regions of the genome have been developed by using γ-irradiated wheat mutants (Komura et al., 2022). These cutting-edge methodologies provide valuable tools for the genetic analyses of the duplicated genome segment(s) in ‘415-1’.

For the practical integration of the mutant ‘415-1’ into pear breeding programs, it is imperative to elucidate the fundamental mechanisms and inheritance patterns of the duplicated *S*-haplotype. In this study, we took a multifaceted approach, encompassing depth-of-coverage analysis through amplicon sequencing targeting *S-RNase* and *SFBB*, ddRAD-seq, and whole-genome resequencing. Its objective was to develop a reliable method that could detect the *S*-haplotype duplication, to estimate the size of the duplicated region(s) within the mutant ‘415-1’, and to clarify the inheritance patterns governing this duplicated segment using offspring seedlings of ‘415-1’.

## 2 Materials and methods

### 2.1 Plant materials and DNA extraction

A population derived from a cross between Japanese pear (*Pyrus pyrifolia* Nakai, 2n =34) breeding lines ‘515-20’ and ‘415-1’ (140 F_1_ seedlings) was used for determination of *S*-haplotype, construction of genetic maps, and investigation of genome duplications. ‘415-1’ was developed by selective fertilization, achieved by pollinating the style of non-irradiated ‘Kosui’ (*S_4_S_5_*) with pollen from γ-irradiated ‘Kosui’ and obtaining a rare successful pollination, indicating the presence of a self-compatibility mutation in the pollen (Sawamura et al., 2013). The *S*-genotype of ‘415-1’ was determined to be *S_4_dS_5_S_5_*, where *d* indicates a partly duplicated haplotype. ‘515-20’ (*S_1_S_3_*) was derived from a cross between ‘Natsushizuku’ and ‘Hatsumaru’ and was among the 6th generation of cultivars produced in the Japanese pear breeding program at the Institute of Fruit Tree and Tea Science, National Agriculture and Food Research Organization. Both lines are homozygous in some regions, a consequence of their origin involving crosses between closely related cultivars.

### 2.2 Determination of seedling S-genotypes by amplicon sequencing

Amplicon sequencing was based on simplified AmpSeq, which uses a mixture of two primer sets (Nishio et al., 2023), the first consisting of tailed target primers, the second of primers that contain flow-cell binding sites, indexes, and tail sequences complementary to those in the first set. Primers for *S-RNase* (Ishimizu et al., 1999) and *SFBB18* were used for the target first-primer sets. To obtain the *SFBB18* primers, we used the *SFBB18* sequences found in apple and Chinese pear (Pratas et al., 2018; Huang et al., 2023) as query sequences, and performed a BLAST search against the reference European pear sequence “Bartlett DH Genome v2.0” (Linsmith et al., 2019) to detect the homologue of *SFBB18* in European pear. A primer set was designed to specifically amplify the detected *SFBB18* sequence. Information on the primer sets and the sequence alignment of *S_1_*, *S_3_*, *S_4_*, and *S_5_* are presented in **Supplementary Table S1** and **Supplementary Figure S1**, respectively. PCR amplification was performed in 10 µL containing 5 µL of 2× Green GoTaq G2 Hot Start Master Mix (Promega, Madison, WI, USA), 0.2 µL of the 1st primer set (1 µM), 1 µL of the 2nd primer set (1 µM), 2.8 µL H_2_O, and 1 µL of genomic DNA (2.5 ng/µL) with an initial denaturation of 94°C for 5 min; 35 cycles of 94°C for 30 s, 60°C for 1 min, and 72°C for 30 s; and a final extension at 72°C for 10 min. The products were mixed equally by volume in a single tube and purified with AMPure XP beads (Beckman Coulter, Inc, Bree, CA, USA) following the AMPure XP PCR Purification protocol. The libraries were sequenced by PE 300-bp sequencing on an Illumina MiSeq platform (Illumina, Inc., San Diego, CA, USA). The reads from the Illumina MiSeq were demultiplexed to each cultivar or seedling on the basis of the index sequences in the 2nd primer set, and paired fastq files of each cultivar were obtained. The fastq files were trimmed of adapter sequences and low-quality bases in Trimmomatic v. 0.39 software (Bolger et al., 2014). The paired fastq files were merged in flash2 software with parameters “-M 150 -X 0.05 –allow-outies” (Magoč and Salzberg, 2011). The merged reads were aligned and stacked to obtain the sequences of *S-RNase* and *SFBB18*. For each marker, the allele frequencies of the three most common haplotypes were calculated for each individual by using a custom script (**Supplementary File 1**).

### 2.3 ddRAD-seq analysis, genetic map construction, and delta depth analysis

Libraries for ddRAD-Seq were constructed as described in Shirasawa et al. (2016). A total of 200 ng of genomic DNA from each seedling or cultivar was double-digested with *Pst*I and *Msp*I (FastDigest restriction enzymes; Thermo Fisher Scientific, Waltham, MA, USA), ligated to adapters in the LigaFast Rapid DNA Ligation System (Promega, Madison, WI, USA), and purified with Agencourt AMPure XP reagent (Beckman Coulter, Brea, CA, USA) to eliminate short (<300 bp) DNA fragments. Purified DNA was diluted with H_2_O and amplified by 20 cycles of PCR with indexed primers. Amplicons were pooled and separated in a BluePippin 1.5% agarose cassette (Sage Science, Beverly, MA, USA), and fragments of 300–900 bp were purified with a Mini Elute Kit (Qiagen). The library was then sequenced on an MGI DNBSEQ-G400 sequencer. The reads were de-multiplexed on the basis of 8-bp dual index sequences. Raw reads were analyzed in Trimmomatic v. 0.39 software (Bolger et al., 2014) to remove reads with poor-quality ends (*Q* < 30). Sequences were aligned to Bartlett DH Genome v2.0 by using the “mem” command of Burrows–Wheeler aligner v. 0.7.17 software (BWA; Li and Durbin, 2009) with default parameters and avoiding multiple-mapping reads. SNPs and indels were mined by following the Genome Analysis Toolkit 4 (GATK4) best practices pipeline (Van der Auwera et al., 2013). GATK’s Base Quality Score Recalibration (BQSR) was implemented to improve the accuracy of variant calling. No SNPs and indels were filtered using QD, MQ, FS, SOR, HaplotypeScore, MQRankSum, or ReadPosRankSum, because we prioritized the analyses of depth of coverage. The GATK’s HaplotypeCaller was used to call variant with options “–max-reads-per-alignment-start 1000” and “–disable-read-filter NotDuplicateReadFilter” to calculate the depth at each locus, because reads starting at the same position above 50 would be downsampled if the default settings were used. To confirm the SNP distribution mapped to Bartlett DH Genome v2.0, following filtering in VCFtools v. 0.1.16 software (parameters: –minDP 20 –maxDP 100 –min-meanDP 30 –max-meanDP 80; Danecek et al., 2011), we visualized the density of SNPs in ChromoMap software (Anand and Rodriguez Lopez, 2022).

The homozygous regions in both parental individuals (‘515-20’ and ‘415-1’) were estimated by examining the SNPs mapped onto Bartlett DH Genome v2.0 within every 200 kb. If a 200-kb region contained no heterozygous SNPs, that region was presumed to be homozygous. The regions that were homozygous in ‘415-1’, in ‘515-20’, and in both ‘415-1’ and ‘515-20’ were visualized in ChromoMap.

For construction of the genetic map, SNPs and indels were filtered in VCFtools v. 0.1.16 software (parameters: –minDP 15–max-alleles 2 –max-missing 0.9 –maf 0.05 –max-maf 0.95). A genetic map was constructed in LepMap3 software (Rastas, 2017). SNPs showing distorted segregation and no information were removed by using the Filtering2 module with parameter dataTolerance = 0.01. The module SeparateChromosomes2 was used to split the retained SNPs into 17 presumed linkage groups with a LOD score limit between 6 and 13 depending on the linkage group. Subsequently, the JoinSingles2All module was used to assign singular markers to the presumed linkage groups. Finally, the OrderMarker2 module was used for each group. To map both male and female informative markers, the option informativeMask=123 was used for the SeparateChromosomes2, JoinSingles2All, and OrderMarker2 modules.

To estimate the duplicated regions in ‘415-1’ and in seedlings derived from the cross of ‘515-20’ and ‘415-1’, differences in depth of coverage (ΔDP) were estimated. SNPs and indels were filtered with VCFtools (parameters: –minDP 20 –maxDP 100 –min-meanDP 30 –max-meanDP 80). VcfR (Knaus and Grünwald, 2017) was used to extract the depth of coverage (DP defined by GATK). ΔDP was calculated by subtracting the depth of coverage of ‘515-20 (diploid)’ from that of each target individual (diploid or partly triploid). The sliding-window average was applied using the R package windowscanr (Tavares, 2021) based on a window size of 20 SNPs and a step size of 10 SNPs. The 95% and 99% confidence intervals (CIs) were calculated as follows. The sliding average of DP was randomly extracted for each individual and ‘515-20’, and ΔDP was calculated by subtracting each moving average of depth of coverage of ‘515-20’ from that of each individual. The operation was performed 10,000 times, and the top 5% and 1% of ΔDP were defined as 95% and 99% CIs, respectively.

### 2.4 Whole-genome sequencing of six individuals

‘415-1’, ‘515-20’, ‘Seedling047’, ‘Seedling053’, ‘Seedling075’, and ‘Kosui’ were used for whole-genome sequencing. PCR-free DNA libraries for 150-bp paired-end DNA sequencing of the six individuals were constructed by using the TruSeq DNA library preparation kit (Illumina). DNA of the six individuals was sequenced on a NovaSeq sequencer. The same GATK best practice pipeline used for ddRAD-seq was used for SNP calling. Different SNP filtering criteria were applied for ΔDP, SNP index, and ΔSNP index analyses.

After SNPs were filtered in VCFtools (–minDP 20 –maxDP 80 –min-meanDP 30 –max-meanDP 70 –max-missing 1), ΔDP was calculated by subtracting the depth of ‘Kosui’ (diploid) from that of each target individual. A sliding-window average of ΔDP based on a window size of 1 Mb and a step size of 0.5 Mb was used to identify the duplicated regions in each individual. To estimate 95% and 99% CIs, the sliding average of depth of coverage was randomly extracted for each individual and ‘Kosui’, and ΔDP was calculated by subtracting each moving average of depth of coverage of ‘Kosui’ from that of each individual. The operation was performed 5,000 times, and the top 5% and 1% of ΔDP were defined as 95% and 99% CIs, respectively.

The SNP index, defined as the ratio of allelic depth of the reference allele to the total depth at each locus, was used for estimating homozygous regions, bias of allele frequencies, and haplotype structures. First, SNPs were filtered by VCFtools with the following options: –minDP 20 –maxDP 80 –min-meanDP 30 –max-meanDP 70 –max-missing 1 –maf 0.05 –max-maf 0.95 –remove-indels –thin 10000. At each locus, the allelic depth for the reference allele and total depth was extracted in vcfR, and the SNP index was calculated and plotted in ggplot2 software (Wilkinson, 2011).

The ΔSNP indexes, defined as the absolute value of the difference between the SNP index of two individuals, were calculated using ‘415-1’, ‘Seedling047’, ‘Seedling053’, and ‘Seedling075’. The SNPs were filtered by VCFtools with following options: –minDP 20 –maxDP 80 –min-meanDP 30 –max-meanDP 70 –max-missing 1 -remove-indels –maf 0.2 –max-maf 0.8. Plotting of the sliding-window average of ΔSNP index was based on a window size of 1 Mb and a step size of 0.5 Mb in the R packages windowscanr and ggplot2.

### 2.5 Plant height evaluation

Seeds resulting from a cross between breeding lines ‘515-20’ and ‘415-1’ were initially sown in Jiffy Pot Strips in January 2022 and later transferred to a nursery in April 2022. The plants were cultivated under natural light and temperature conditions. In December 2022, when shoot elongation had ceased, the height of one-year-old seedlings was measured from the soil level to the tip of the meristem. Significance in plant height between normal diploid seedlings and partly triploid seedlings was assessed using a one-way analysis of variance (ANOVA) test.

## 3 Results

### 3.1 Observed segregation based on amplicon sequences of seedlings derived from crossing breeding lines ‘515-20’ (S_1_S_3_) and ‘415-1’ (S_4_dS_5_S_5_)

To identify *S*-genotypes and haplotype duplication, we conducted amplicon-sequence-based genotyping for *S-RNase* and *SFBB18* using ‘515-20’, ‘415-1’, and their progeny seedlings (**Supplementary Table S2**). The respective allele frequencies of *S_1_* and *S_3_* in ‘515-20’ (*S_1_S_3_*) were 0.40 and 0.52 for *S-RNase* and 0.42 and 0.49 for *SFBB18*, whereas those of *S_4_* and *S_5_* in ‘415-1’ (*S_4_dS_5_S_5_*) were 0.13 and 0.79 for *S-RNase* and 0.26 and 0.61 for *SFBB18* (**Supplementary Table S2**). Those results support that ‘515-20’ has genotype *S_1_S_3_* and that ‘415-1’ has genotype *S_4_dS_5_S_5_*. Because amplicons of *S-RNase* included more SNPs and indels among *S*-haplotypes than did those of *SFBB18* (**Supplementary Figure S1**), amplification bias among alleles was higher for *S-RNase* than for *SFBB18.* For example, the ratios of *S_5_*/*S_4_* frequencies were 6.20 in *S-RNase* and 2.39 in *SFBB18*, though the expected ratio is 2.00 for ‘415-1’ because of the duplicated *S_5_* region. Likewise, the *S_3_*/*S_1_* ratio and for ‘515-20’ was 1.32 in *S-RNase* and 1.15 in *SFBB18* (the expected ratio was 1.00). Because of the smaller allele bias in *SFBB18*, we used the *SFBB18* amplicon to detect *S*-haplotype duplication. Among the seedlings derived from ‘515-20’ and ‘415-1’, 22 out of 140 were determined to have third alleles, because the frequency of the third *SFBB18* allele was ≥0.2 in those seedlings. In the seedlings in which only two alleles were detected and the ratio of first/second allele frequencies exceeded 1.5, seedlings were determined to carry a duplication of the first allele and thus to show a partially triploid genotype (e.g., *S_3_S_4_dS_4_* in Seedling053 and *S_3_S_5_dS_5_* in Seedling075) (**Supplementary Table S2**).

The segregation estimated from the amplicon sequencing of *SFBB18* is summarized in **Table 1**. Interestingly, 30 seedlings with triploid genotypes—*S_1_S_4_dS_5_* (7 seedlings), *S_3_S_4_dS_5_* (15), *S_1_S_5_dS_5_* (3), *S_3_S_5_dS_5_* (4), and *S_3_S_4_dS_4_* (1)—were detected out of the 140 seedlings derived from ‘515-20’ × ‘415-1’. The segregation of female gametes derived from ‘515-20’ was *S_1_*:*S_3 =_* 72:68, which is not significantly different from the 1:1 ratio expected for monogenic inheritance. On the other hand, the segregation of male gametes derived from ‘415-1’ was *S_5_*:*S_5_dS_5_*:*S_4_*:*S_4_dS_4_*:*S_4_dS_5 =_* 107:7:3:1:22, which is presumed to have deviated from Mendelian inheritance. Direct genotyping of single pollen grains in a previous study showed *S_4_*:*S_5_*:*S_4_dS_5 =_* 10:135:28 for *S-RNase* frequency (Mase et al., 2014b). As that study could not detect allele duplications (i.e., *S_5_dS_5_* and *S_4_dS_4_* gametes were presumed to be included with *S_5_* and *S_4_* gametes, respectively), the results obtained here and in Mase et al. (2014b) are similar. The microscopic analysis revealed that ‘415-1’ contained sterile pollen with a smaller size compared to ‘Kosui’ (Mase et al., 2014b). The abortion of pollen likely results from abnormal meiosis during gamete formation caused by genome duplication, leading to significant segregation distortion in male gametes.

**Table 1:** Number of individuals in combinations of each gamete derived from crossing 515-20 and 415-1.

		Male gamete derived from 415-1 (*S_4_dS_5_S_5_*)					*S*-genotype
		*S_5_*	*S_5_dS_5_*	*S_4_*	*S_4_dS_4_*	*S_4_dS_5_*	
Female gamete derived from 515-20 (*S_1_S_3_*)	*S_1_*	60					*S_1_S_5_*
			3				*S_1_S_5_dS_5_*
				2			*S_1_S_4_*
					0		*S_1_S_4_dS_4_*
						7	*S_1_S_4_dS_5_*
	*S_3_*	47					*S_3_S_5_*
			4				*S_3_S_5_dS_5_*
				1			*S_3_S_4_*
					1		*S_3_S_4_dS_4_*
						15	*S_3_S_4_dS_5_*

### 3.2 ddRAD genotyping and constructing a genetic map

**Figure 1:**
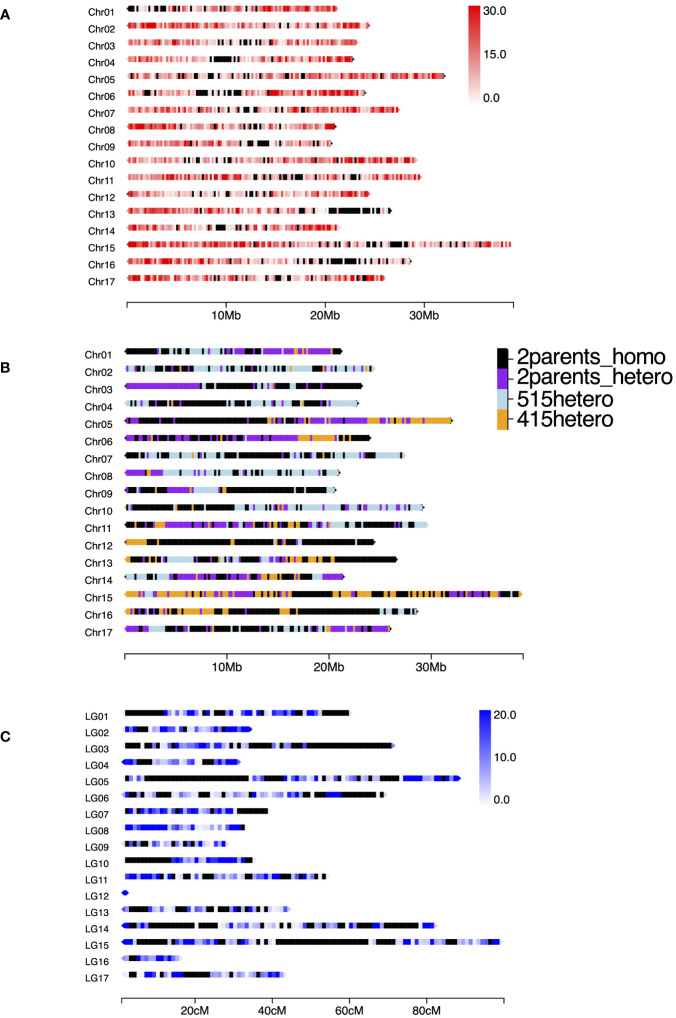
**(A)** Density of SNPs mapped to *Pyrus communis* Bartlett DH Genome v2.0 by ddRAD-seq using a population derived from a cross between ‘515-20’ and ‘415-1’. In total, 28,117 SNPs were obtained through the GATK best practice pipeline after filtering with mean minimum depth of 30. The window size was set to 200 kb. The heat map indicates the number of SNPs per 200-kb region; black windows indicate regions in which no SNPs were mapped. **(B)** Illustration of homozygous and heterozygous regions in ‘515-20’ and ‘415-1’. Labels: “2parents_homo”, homozygous in ‘515-20’ and ‘415-1’; “2parents_hetero”, heterozygous in ‘515-20’ and ‘415-1’; “515hetero”, heterozygous in only ‘515-20’; “415hetero”, heterozygous in only ‘415-1’. **(C)** SNP density based on the linkage map constructed by LEPMAP3 (cM). The window size was set to 1 cM; black windows indicate regions in which no SNPs were mapped. The number of SNPs mapped to the consensus map of ‘415-1’ and ‘515-20’ was 6,890.

In total, 247.9 M reads were obtained from the MGI DNBSEQ-G400 sequencer. The numbers of reads per sample ranged from 1.09 M to 2.86 M with an average of 1.77 M. After cleaning and trimming, the retained sequences were mapped to Bartlett DH Genome v2.0, and 433,922 SNPs and indels were obtained through the GATK best practices pipeline. To confirm SNP density mapped on the reference map, we extracted 27,554 SNPs that showed mean depth of >30× and counted them in each 200 kb (**Figure 1A**). Except for several regions in which no SNPs were mapped, the SNPs were almost equally distributed among the chromosomes. Because recent breeding lines, including ‘515-20’ and ‘415-1’, were developed from ‘Nijisseiki’ and its relatives (Nishio et al., 2016), those lines include some homozygous regions. Before constructing a genetic map, we determined the homozygous regions of ‘515-20’ and ‘415-1’. When a parent had at least one heterozygous SNP in a given 200-kb region, the parent was classified as heterozygous in that region. Interestingly, 46.1% of the genome was homozygous in both ‘515-20’ and ‘415-1’ (**Figure 1B**), 19.9% was heterozygous in both, 12.3% was heterozygous in only ‘515-20’, and 21.7% was heterozygous in only ‘415-1’. After removal of SNPs that had >10% missing data, <15 depth, and <0.05 allele frequency, 11,451 SNPs were retained and used to construct a consensus genetic map of ‘515-20’ and ‘415-1’. The 17 linkage groups were created in accordance with the order of Bartlett DH Genome v2.0 with 6,890 SNPs (**Figure 1C**). The total length of the map was 810 cM, which was smaller than the consensus maps in other studies (1,433 to 3,266 cM; Wu et al., 2014; Yamamoto et al., 2014; Chen et al., 2015; Li et al., 2017; Gabay et al., 2018; Nishio et al., 2018). The lengths of chromosomes 12 and 16 were 1.8 and 15.2 cM, respectively, owing to the long homozygous regions from both parents, which were non-informative for constructing the map. There were also some gaps in several regions, including the first parts of chromosomes 01 and 10, the middle part of chromosome 05, and the end parts of chromosomes 03 and 15. Those regions also corresponded to those that were homozygous in both ‘515-20’ and ‘415-1’ (**Figure 1B**).

### 3.3 Delta DP analyses using ddRAD-seq

**Figure 2:**
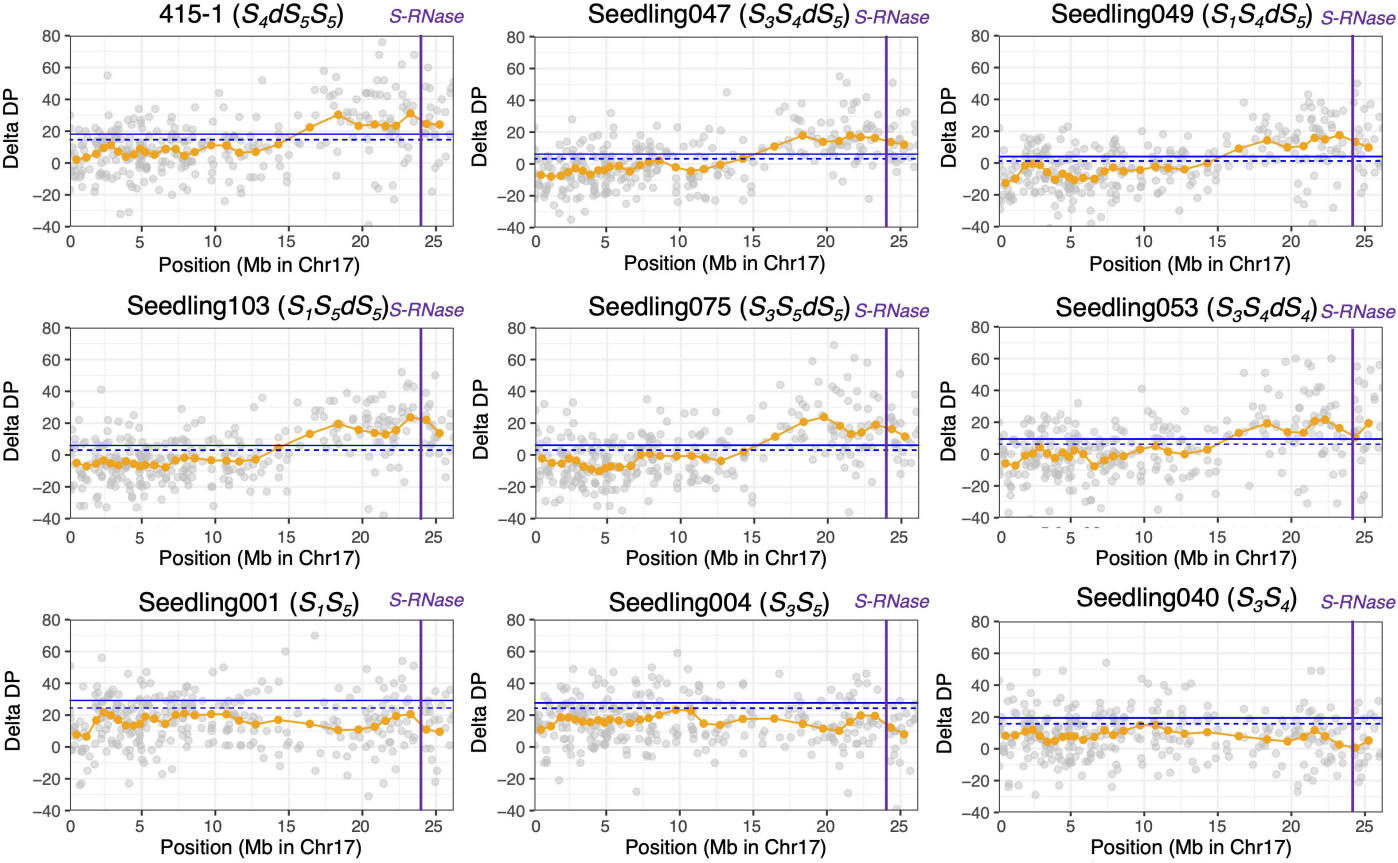
ΔDP analysis using SNPs obtained through ddRAD-seq of chromosome 17. The depth of coverage of each individual was subtracted from that of parental individual ‘515-20’. Gray circles indicate the ΔDP at each locus, and orange circles indicate the sliding-window average based on a window size of 20 SNPs and a step size of 10 SNPs. The blue horizontal line (upper), 99% CI; blue dotted horizontal line (lower), 95% CI; purple vertical lines, position of *S-RNase*. Nine individuals with different *S*-genotypes are represented, but the other individuals that had the same *S*-genotype showed similar ΔDP patterns.

Before conducting ΔDP analyses, we extracted the SNPs that showed an average depth of 30 to 80. The average depth of all filtered SNPs from the breeding lines and seedlings was 48.1. The read depths of ‘415-1’ and progeny seedlings were subtracted from those of ‘515-20’, and the sliding-window average based on a window size of 20 SNPs was used to confirm the duplicated region. The plot of ΔDP of ‘415-1’ showed a long duplicated region from positions 15 Mb to 26.5 Mb on chromosome 17 (**Supplementary Figure S2**). Although the delta depth with window size of 20 SNPs around the 22-Mb position of chromosome 2 in ‘415-1’ exceeded the 99% CI, most other regions, including those on other chromosomes, had no duplicated regions. The plots of ΔDP on chromosome 17 of ‘415-1’ and eight progeny seedlings are shown in **Figure 2**. The seedlings determined to carry three *S*-haplotypes by amplicon sequencing targeting *SFBB18* and *S-RNase* have a long genome duplication in the region from 15 to 26.5 Mb on chromosome 17. Interestingly, the length of the duplicated region in ‘415-1’ and its progeny that had three *S*-haplotypes was constant, indicating that the duplicated region maintained a certain length over generations. Results from only eight progeny seedlings (representing eight different *S*-genotypes) are plotted in **Figure 2**, but the other 132 individuals showed patterns on chromosome 17 similar to those that had the same *S*-genotype.

### 3.4 Whole-genome sequence analysis using six individuals

**Figure 3:**
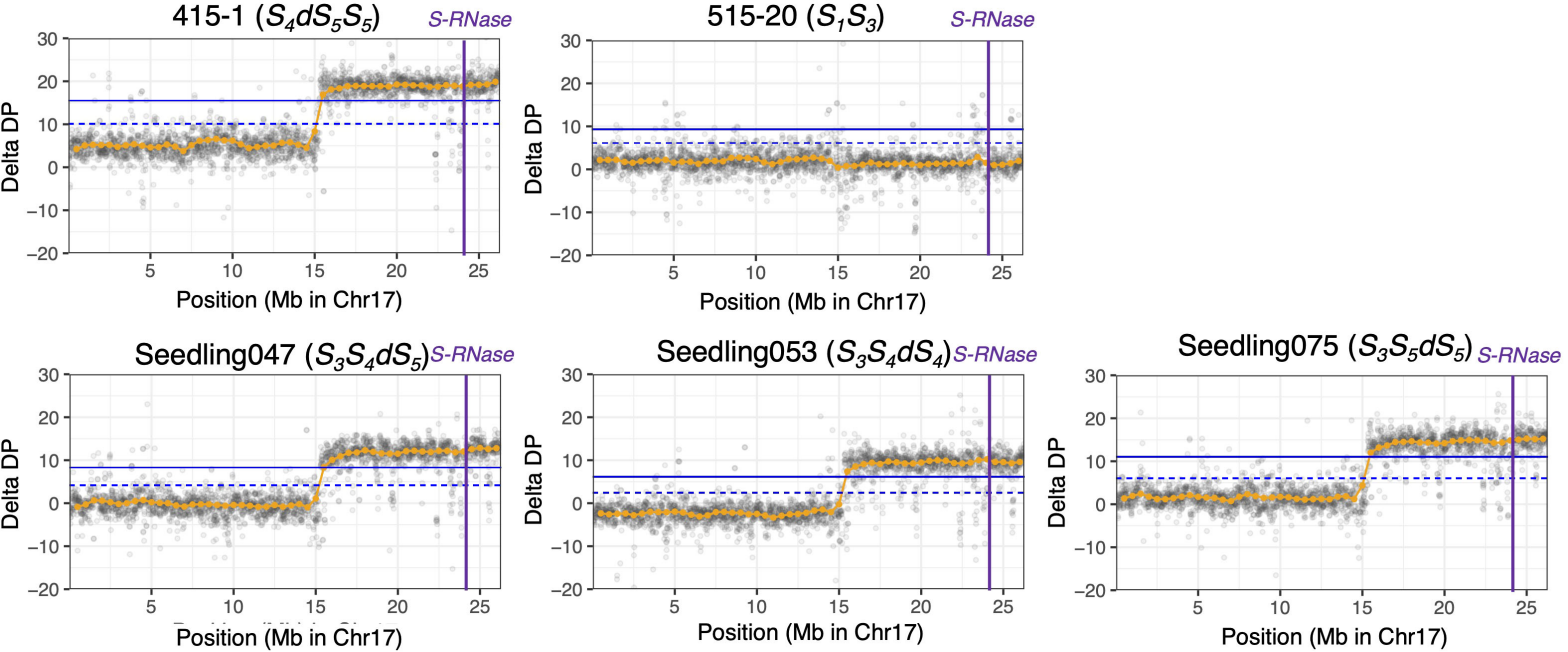
ΔDP analysis using whole-genome resequencing data for chromosome 17. The depth of coverage of each individual was subtracted from that of ‘Kosui’. Orange points indicate sliding-window averages of ΔDP based on a window size of 1 Mb and a step size of 0.5 Mb. Gray points indicate sliding-window averages of ΔDP based on a window size of 20 kb and a step size of 10 kb. Blue horizontal line(upper), 99% CI; blue dotted horizontal line (lower), 95% CI; purple vertical lines, position of *S-RNase*.

Using PCR-free DNA libraries for 150-bp paired-end DNA sequencing, we obtained 140 M to 168 M total reads for ‘415-1’, ‘515-20’, ‘Kosui’, and three seedlings representing different triploid genotypes (‘Seedling047’, ‘Seedling053’, and ‘Seedling075’; **Table 2**). After trimming and cleaning of whole-genome sequence data, we mapped 135 M to 164 M reads to Bartlett DH Genome v2.0, representing 40.7×to 49.5× genome depth. Variant calling obtained 18,716,743 SNPs for the six individuals. After selecting of SNPs with a mean depth of 30 to 70, 4,867,289 SNPs were retained. ΔDP analyses using whole-genome sequencing showed results similar to those obtained by ddRAD-seq (**Supplementary Figures S2**, **Figure 3**). The limits of the duplicated region on chromosome 17 was estimated more accurately than with ddRAD-seq, and ranged from positions 15.3 Mb to 26.3 Mb in ‘415-1’ (**Supplementary Figure S3**), ‘Seedling047’, ‘Seedling053’, and ‘Seedling075’, all of which had three *S*-haplotypes, whereas ‘515-20’ did not show any duplication anywhere in the genome (**Supplementary Figure S4**). Whole-genome delta depth analysis did not detect any other duplicated or deleted region in ‘415-1’, ‘Seedling047’, ‘Seedling053’, and ‘Seedling075’ (**Supplementary Figure S4**), including the region around 22 Mb on chromosome 2, where ΔDP of ddRAD-seq detected a duplication (**Supplementary Figure S2**). Considering the larger number of markers used for estimating ΔDP for whole-genome sequencing than for ddRAD, we conclude that the genome duplication was limited to an 11-Mb region of chromosome 17.

**Table 2:** Summary of the whole-genome sequences of six individuals used in this study.

Individual	Number of total reads	Total reads (Gb)	Number of cleaned reads	Number of reads mapped to Bartlett DH Genome v2.0	Total mapped reads (Gb)	Q20 (%)	Q30 (%)	GC (%)	Average depth of coverage
415-1	168,467,380	25.3	166,570,886	164,086,057	24.6	96.9	91.6	37.9	49.5
515-20	161,263,742	24.2	159,163,368	157,009,244	23.6	96.7	91.3	37.7	47.4
Kosui	153,114,240	23.0	151,114,928	149,043,972	22.4	96.6	91.0	37.9	45.0
Seedling047	140,565,228	21.1	138,943,604	137,133,459	20.6	97.0	91.8	37.6	41.4
Seedling053	139,843,208	21.0	137,754,770	134,899,112	20.2	96.5	90.8	37.8	40.7
Seedling075	149,713,056	22.5	147,683,034	145,748,688	21.9	96.8	91.4	37.5	44.0

**Figure 4:**
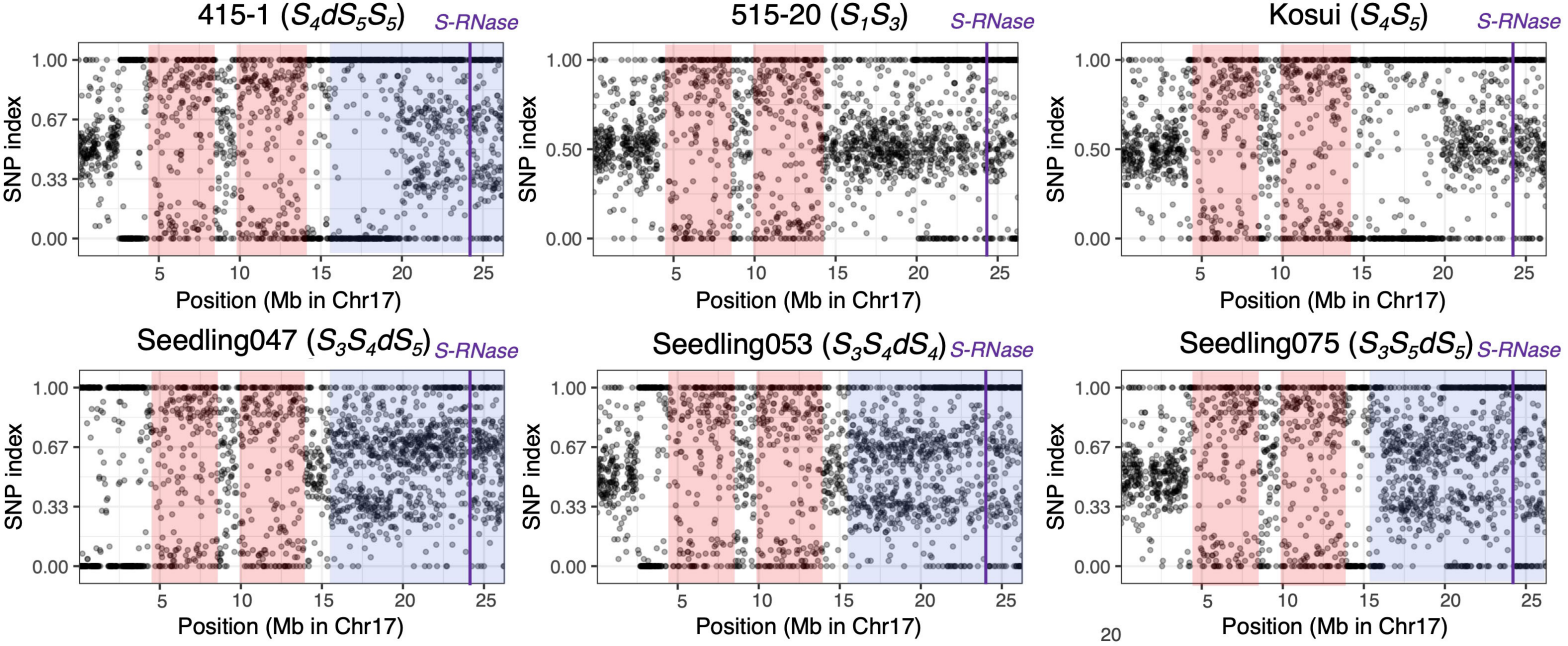
Allele frequencies covering a given SNP position in chromosome 17 of six individuals. At each locus, the SNP index was defined as the ratio of allelic depth of the reference allele to the total depth at that locus. The 1,902 SNPs were chosen by extracting one SNP per kb. Pink shading indicates homozygous regions that are common in the six individuals. Blue shading indicates putative triploid regions. An SNP index around 0.5 indicates a heterozygous diploid region. SNP indexes around 0.33 and 0.67 in partially triploid regions indicate that the locus carries one reference and two alternative alleles, or two reference and one alternative alleles, respectively. Purple vertical lines indicate position of *S-RNase*.

To detect homozygous regions and to confirm the duplicated region on chromosome 17, we calculated the SNP indexes for the six individuals used in the whole-genome sequence analysis (**Figure 4**). The distribution of SNP indexes showed that there were two homozygous regions common to all six individuals, including positions 4.0–8.2 Mb and 9.8–14.0 Mb. In ‘415-1’, those homozygous regions extended to positions 2.6–8.5 Mb and 9.8–19.6 Mb, and the region from position 15.3 Mb to 19.6 Mb overlapped with the duplicated region. In ‘Seedling047’, ‘Seedling053’, and ‘Seedling075’, the SNP index of position 15.3–26.3 Mb on chromosome 17 was around either 0.33 or 0.67, indicating that this region was triploid, with each locus carrying either one reference allele and two alternative alleles or two reference alleles and one alternative allele.

**Figure 5:**
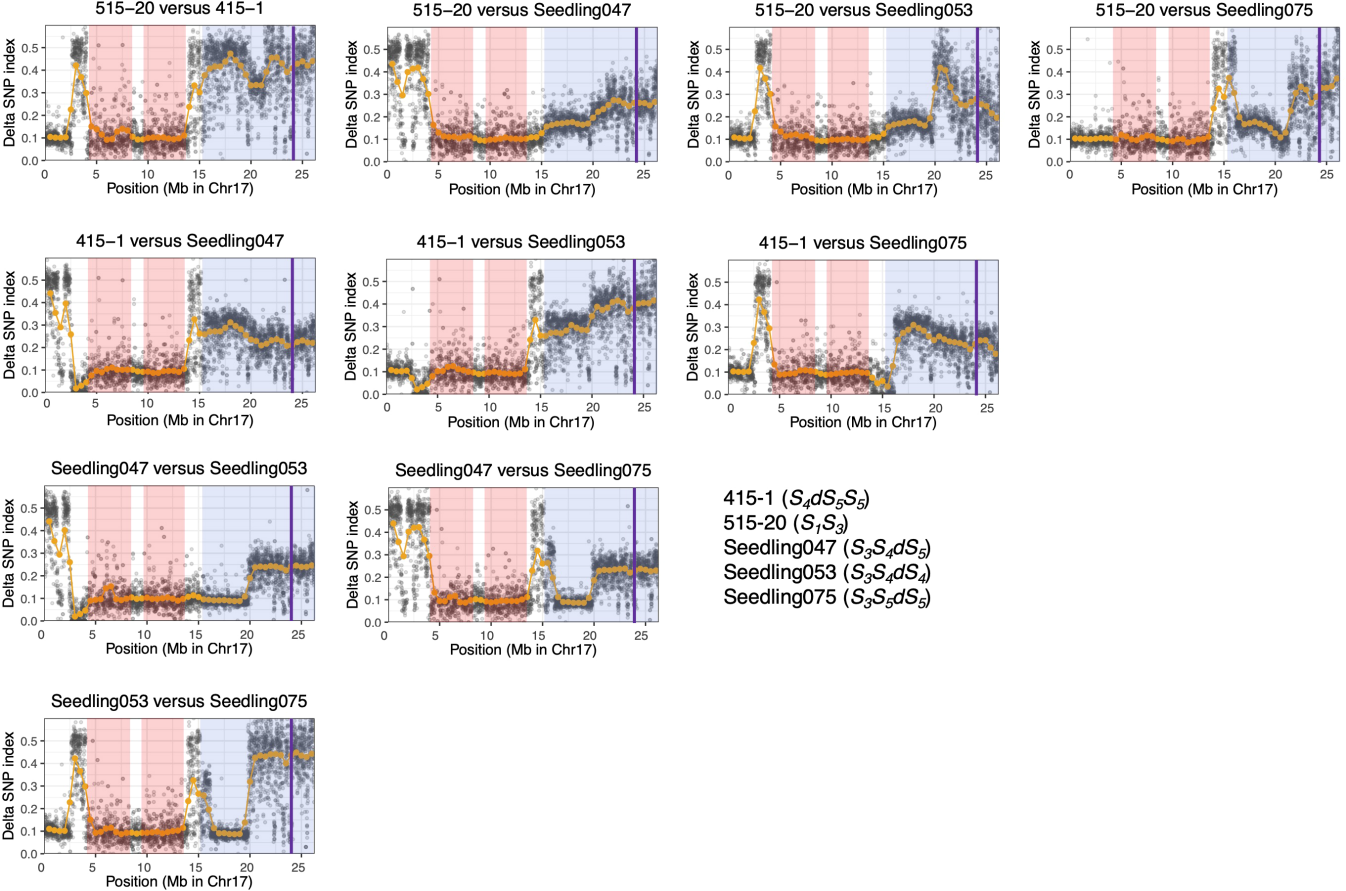
ΔSNP index analyses using ‘415-1’, ‘Seedling047’, ‘Seedling053’, and ‘Seedling075’. At each locus, the SNP index was defined as the ratio of allelic depth of the reference allele to the total depth at that locus, and the ΔSNP index was defined as the absolute value of the difference between the SNP indexes of each pair of individuals. Orange points indicate sliding-window average of ΔSNP index based on a window size of 1 Mb and a step size of 0.5 Mb. Gray points indicate sliding-window average of ΔSNP index based on a window size of 10 kb and a step size of 5 kb. A ΔSNP index around 0.1 indicates that the two individuals share the same genotype at a given locus. Pink shading indicates homozygous regions that are common in the six individuals. Blue shading indicates putative triploid regions. Purple vertical lines indicate the position of *S-RNase*.

**Figure 6:**
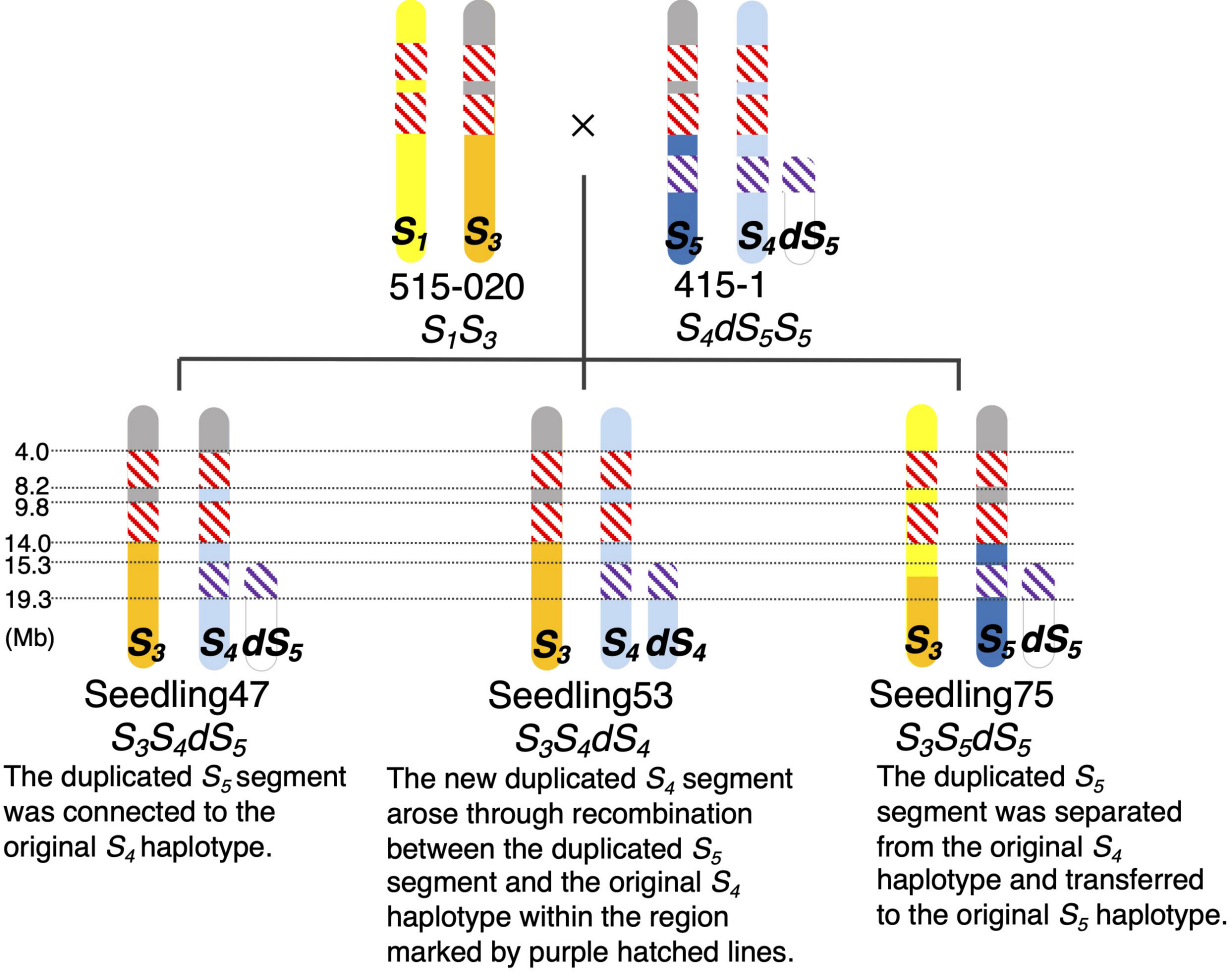
Putative structures of chromosome 17 of ‘515-20’, ‘415-1’, ‘Seedling047’, ‘Seedling053’, and ‘Seedling075’. Colors indicate chromosome regions originating from the parental chromosomes containing (yellow) *S_1_*, (orange) *S_3_*, (blue) *S_5_*, (light blue) *S_4_*, and (white) duplicated *S_5_* (*dS_5_*). Gray: upper regions of *S_5_* and *S_3_* had common haplotypes. Hatching: red, homozygous regions common among the five individuals; purple, region common in the *S_5_*, *S_4_*, and *dS_4_* chromosome regions derived from ‘415-1’. Although there were some other common haplotype regions <3 Mb, such as between 8.2 and 9.8 Mb in parental chromosomes *S1* and *S4*, those regions are shaded according to their specific parental chromosomes to simplify the illustration.

We calculated ΔSNP indexes to infer regions identical among ‘515-20’, ‘415-1’, ‘Seedling047’, ‘Seedling053’, and ‘Seedling075’ (**Figure 5**), which would help to clarify the haplotype structure around the duplicated region on chromosome 17. On the basis of the ΔSNPs and ΔSNP indexes, we constructed putative haplotype structures of ‘415-1’, ‘515-20’, ‘Seedling047’, ‘Seedling053’, and ‘Seedling075’ (**Figure 6**). All five individuals shared the same genotypes at positions 4.0–14.0 Mb on chromosome 17, while some of them had different genotypes at positions 0.0–4.0 Mb and 14.0–15.3 Mb. In duplicated regions, all five individuals had different genotypes at position 19.3–26.3 Mb, in accordance with their having different *S*-genotypes (*S_1_S_3_*, *S_4_dS_5_S_5_*, *S_3_S_4_dS_5_*, *S_3_S_4_dS_4_*, and *S_3_S_5_dS_5_*, respectively). On the other hand, ‘Seedling047’, ‘Seedling053’, and ‘Seedling075’ shared the same genotypes at position 16.3–19.3 Mb, whereas they had different genotypes at position 15.3–16.3 Mb. The result suggests a recombination event between *S_1_* and *S_3_* haplotypes at around position 16.3 Mb in a gamete giving rise to ‘Seedling075’ (**Figure 6**).

The inheritance of duplicated segments in ‘Seedling047’, ‘Seedling053’, and ‘Seedling075’ was estimated as follows (**Figure 6**). ‘Seedling047’ had an *S_3_S_4_dS_5_* genotype, and the duplicated *S_5_* segment would have been connected to the original *S_4_* haplotype of ‘415-1’. ‘Seedling053’ had an *S_3_S_4_dS_4_* genotype, in which the new duplicated *S_4_* segment would have been developed through recombination between the duplicated *S_5_* segment and the original *S_4_* haplotype. ‘Seedling075’ had an *S_3_S_5_dS_5_* genotype, indicating that the duplicated *S_5_* segment was transferred from the original *S_4_* haplotype to the original *S_5_* haplotype.

### 3.5 Plant height evaluation

The average height of normal diploid seedlings was 149.3 cm, whereas partly triploid individuals measured 136.2 cm (**Supplementary Table S3**), with no significant difference found by ANOVA. Although some partly triploids tended to germinate later, there were no clear distinctions observed between diploids and triploids in subsequent growth, including tree vigor and stem diameter.

## 4 Discussion

### 4.1 Using amplicon sequencing targeting S-RNase and SFBB to detect the duplicated segment

Several methods exist to determine *S*-genotype, including allele-specific PCR and PCR-RFLP (Ishimizu et al., 1999; Takasaki et al., 2004; Kakui et al., 2007; Mase et al., 2014a; Nashima et al., 2015). However, none of these methods can detect *S*-gene duplication. Here, we determined the *S*-genotype of individuals, including partly triploid individuals, using amplicon sequencing targeting *S-RNase* and *SFBB18*. This method can detect allele duplication effectively. Notably, it revealed novel genotypes that were not identified by the previous method (Mase et al., 2014a)—including *S_3_S_4_dS_4_*, *S_3_S_5_S_5_*, and *S_1_S_5_S_5_*—among seedlings derived from crossing ‘515-20’ (*S_1_S_3_*) and ‘415-1’ (*S_4_dS_5_S_5_*). We observed minimal bias in amplification of the *SFBB18* amplicon, allowing us to confirm allele duplications with a high degree of precision. Nevertheless, it is worth noting that *SFBB18* loci are positioned at 448 kb from the *S-RNase* locus in the genome of ‘Bartlett’ and 264 kb in the genome of ‘Nijisseiki’ (Shirasawa et al., 2021). The detection of one seedling exhibiting recombination between *SFBB18* and *S-RNase* (‘Seedling059’; **Supplementary Table S2**), evidenced by the fact that *SFBB18* had one genotype (*S_1_S_5_*) and *S-RNase* had another (*S_3_S_5_*), implies that *SFBB18* likely lies outside the *S*-haplotype. Similar to the findings of this study, some *SFBB* genes in European pear also exhibited an incomplete linkage to the *S-RNase* (de Franceschi et al., 2011). These genes and *SFBB18* in this study would have placed in close proximity of *S*-haplotype but not belonging to it. It is possible that some *SFBB* genes were escaped from *S*-haplotype in the long term evolution of S-locus.

Due to the presence of recombination between *SFBB18* and *S-RNase*, it is better to use *S-RNase* for determination of the genotype of the *S*-haplotype in normal diploid cultivars. On the other hand, our method using both *SFBB18* and *S-RNase* amplicons would be preferable for marker-assisted selection of self-compatibility in practical breeding programs because it is useful for detecting duplicated loci. Individuals carrying three distinct alleles (*S_3_S_4_dS_5_*, *S_1_S_4_dS_5_*) are typically self-compatible. However, it is noteworthy that partially triploid individuals with genotypes such as *S_1_S_4_dS_4_*, *S_3_S_4_dS_4_*, *S_1_S_5_dS_5_*, and *S_3_S_5_dS_5_* may not necessarily be self-compatible, as they may not produce heteroallelic pollen (e.g., *S_1_dS_4_*, *S_3_dS_4_*, *S_1_dS_5_*, and *S_3_dS_5_*), which is needed to break down self-incompatibility by detoxification of non-self S-RNase proteins. In other words, pollen carrying the same two alleles (*S_4_dS_4_* pollen and *S_5_dS_5_* pollen) would be rejected by their *S-RNase* counterparts (*S_4_* and *S_5_*, respectively) in the pistil.

### 4.2 Depth of coverage using ddRAD and whole-genome sequencing clarified the duplicated region

Whole-genome sequencing analyses using sliding-window techniques have found extensive application in quantitative trait locus sequencing (QTL-seq), employing both SNP index and ΔSNP index methodologies (Takagi et al., 2013; Xue et al., 2017; Yamakawa et al., 2021). Furthermore, investigations of genome duplications and deletions within γ-irradiated mutants have used depth-of-coverage analysis (Komura et al., 2022). This approach facilitates the identification of relatively large genomic duplications and deletions by subtracting the depth of coverage observed in mutants from that of the original cultivar, within a sliding-window framework. We showed here that this method works well for pear, a heterozygous perennial crop. And we modified it to be applicable to sequence data obtained by ddRAD-seq. For sliding-window analysis in ddRAD-seq, instead of using a physical distance of 200 kb, we defined a region as including 20 SNPs. This modification makes it easier and more cost-effective to detect duplicated regions in a large number of individuals.

### 4.3 The size and inheritance of the duplicated segments

Mutations produced by γ-irradiation include insertions, deletions, translocations, duplications, and structural variations (Komura et al., 2022; Abe et al., 2023; Hase et al., 2023). Because ‘415-1’ arose through pollination of a self-incompatible cultivar (‘Kosui’) with γ-irradiated pollen of the same cultivar, a mutation in the *S*-haplotype was presumed (Sawamura et al., 2013). The duplication around *S*-haplotypes in ‘415-1’ was substantiated by the detection of a third set of alleles for *RNase*, *SFBB^−γ^*, and the flanking SSRs (Mase et al., 2014a). This finding was further corroborated through single-pollen genotyping techniques (Mase et al., 2014b). In this study, we clarified that the duplicated segment in ‘415-1’ spanned ~ 11.0 Mb, considerably larger than anticipated, and that it remained consistent in size when inherited by partially triploid progeny. In most cases, the original (non-mutant) *S_4_* segment was transmitted to the next generation in combination with the duplicated *S_5_* segment. But the duplicated *S_5_* segment was sometimes lost from association with the *S_4_* original haplotype, resulting in transmission of only the original *S_4_* haplotype to the next generation. This separation of the original *S_4_* haplotype and the duplicated *S_5_* segment was also confirmed by single-pollen genotyping (Mase et al., 2014b). Interestingly, recombination between the duplicated *S_5_* segment and the original *S_4_* haplotype appears to have occurred in a gamete giving rise to ‘Seedling053’ (*S_3_S_4_dS_4_*), suggesting that the duplicated segment participated in pairing and recombination despite being less than half the size of a typical chromosome 17. Because other chromosomes did not show any duplication or deletion in ‘415-1’ or its progeny, the duplicated segment remained in chromosome 17 (i.e., it had not been translocated to another chromosome). The normal appearance of the genetic linkage map constructed using ddRAD-seq also supports the absence of translocations involving the duplicated segment.

Given the pivotal role of centromeres in mitosis and meiosis (Pluta et al., 1995), it is reasonable to infer that this duplicated region encompasses the centromeric region. Triploids in plant species often arise from the fusion of a reduced gamete (*n*) and an unreduced gamete (2*n*) (Pelé et al., 2018; Howard et al., 2023). Unreduced gametes are produced by failure of disjunction of homologous chromosomes in meiosis. There are two types of nondisjunction: first- and second-division restitution (FDR and SDR, respectively). The nondisjunction in FDR produces heterozygous gametes that may accompany a recombination event, whereas that in SDR produces homozygous gametes. Since ‘415-1’ seemed to carry heterozygous gametes (*S_4_* and *S_5_*), the duplication may be derived from FDR.

### 4.4 The future use of partially triploid seedlings in pear breeding programs

Within the Pyreae tribe, the occurrence of triploid cultivars has been well documented in various fruit species, including apple, European pear, and loquat (Watanabe et al., 2008; Ferreira dos Santos et al., 2011; He et al., 2012; Lassois et al., 2016; Larsen et al., 2017; Baccichet et al., 2020). Among Asian pears, relatively few triploid accessions have been identified (Kadota and Niimi, 2004; Postman et al., 2015), despite their advantage of self-compatibility. The scarcity of triploid Asian pear cultivars is likely due to challenges related to pollen and seed fertility and overall viability. In the case of ‘415-1’ and its progeny, the triploid region of chromosome 17 is limited to an 11-Mb segment, and this limitation may help to overcome issues with reduced fertility and viability often associated with triploids. In fact, we did not observe significant differences in plant height between partially triploid and diploid seedlings (**Supplementary Table S3**). ‘415-1’ and these partly triploid seedlings will be useful materials for Asian pear breeding programs. An efficient method for obtaining self-compatible seedlings is selective pollination using partially triploid individuals to pollinate diploid cultivars that share common *S-*haplotypes. For example, pairing an *S_4_dS_5_S_5_* pollen parent and an *S_4_S_5_* seed parent can yield self-compatible offspring without the need for marker-assisted selection techniques.

## 5 Conclusion

This study presents a reliable method for identifying *S*-genotypes in partially triploid pears by using amplicon sequencing. Such identification is crucial for breeding programs. In addition, whole-genome sequencing analyses with sliding-window approaches elucidated the 11.0-Mb duplication on chromosome 17 in partially triploid individuals descended from ‘515-20’ × ‘415-1’. The duplicated segment functions as a chromosome, because recombination between the 11.0-Mb duplicated segment and the original chromosome was observed in a *S_3_S_4_dS_4_* seedling. The inheritance of these duplicated segments by subsequent generations of seedlings occurs at a consistent rate and individuals that produce heteroallelic pollen would acquire self-compatibility. This research and the partially triploid individuals developed here offer valuable insights and practical applications for pear breeding and genetics.

## Data availability statement

The datasets presented in this study can be found in online repositories. The names of the repository/repositories and accession number(s) can be found below: Sequence Read Archive (DRA) of DNA Data Bank of Japan (DDBJ) under accession numbers DRA016310 and DRA016311.

## Author contributions

SN: Conceptualization, Formal analysis, Funding acquisition, Investigation, Methodology, Writing – original draft. KS: Methodology, Writing – review & editing. RN: Methodology, Writing – review & editing. YT: Investigation, Resources, Writing – review & editing. AI: Investigation, Resources, Writing – review & editing. NM: Investigation, Resources, Writing – review & editing. NT: Investigation, Resources, Writing – review & editing.
